# Recent Progress in Developing Extracellular Vesicles as Nanovehicles to Deliver Carbohydrate-Based Therapeutics and Vaccines

**DOI:** 10.3390/vaccines13030285

**Published:** 2025-03-07

**Authors:** Japigorn Puagsopa, Niksa Tongviseskul, Thapakorn Jaroentomeechai, Bunyarit Meksiriporn

**Affiliations:** 1 Department of Physiology and Aging, College of Medicine, University of Florida, Gainesville, FL 32610, USA; jpuagsopa@ufl.edu; 2 Department of Biology, School of Science, King Mongkut’s Institute of Technology Ladkrabang, Bangkok 10520, Thailand; 65050319@kmitl.ac.th; 3 Copenhagen Center for Glycomics, Departments of Cellular and Molecular Medicine, Faculty of Health Sciences, University of Copenhagen, Blegdamsvej 3, 2200 Copenhagen, Denmark

**Keywords:** extracellular vesicles, glycoconjugate vaccines, glycan drug delivery, Alzheimer’s disease, cancer therapy, synthetic glycobiology

## Abstract

Cell-derived, nanoscale extracellular vesicles (EVs) have emerged as promising tools in diagnostic, therapeutic, and vaccine applications. Their unique properties including the capability to encapsulate diverse molecular cargo as well as the versatility in surface functionalization make them ideal candidates for safe and effective vehicles to deliver a range of biomolecules including gene editing cassettes, therapeutic proteins, glycans, and glycoconjugate vaccines. In this review, we discuss recent advances in the development of EVs derived from mammalian and bacterial cells for use in a delivery of carbohydrate-based protein therapeutics and vaccines. We highlight key innovations in EVs’ molecular design, characterization, and deployment for treating diseases including Alzheimer’s disease, infectious diseases, and cancers. We discuss challenges for their clinical translation and provide perspectives for future development of EVs within biopharmaceutical research and the clinical translation landscape.

## 1. Introduction

Extracellular vesicles (EVs) are lipid bilayer-based particles released from cells. EVs are heterogeneous in size, ranging from 30 nm to 10 µm in diameter. They are composed of biomolecules including cellular proteins, surface receptors, free fatty acids, and nucleic acids as well as various metabolites derived from parental cells [1,2,3]. Studies have shown that EVs are critical in regulating some physiological processes and, in certain circumstances, they contribute to the pathological transformation within the human body. As natural molecular cargo, EVs can facilitate cell–cell communication, promote cell proliferation, support tissue regeneration and angiogenesis, modulate the immune system, and mediate host–microbe interactions [4,5,6].

Extracellular vesicles (EVs) are naturally produced by both eukaryotic and prokaryotic cells. To date, mammalian and bacterial EVs are the most extensively studied EVs. In humans, EVs are released from various cell types, including induced pluripotent stem cells, neuronal stem cells, and immune cells (e.g., macrophages, neutrophils, dendritic cells, and lymphocytes), and these EVs play crucial roles in modulating immune responses, including activation, suppression, and intercellular communication [7,8]. Similarly, Gram-negative and Gram-positive bacteria secrete nano-structured membrane vesicles into the extracellular environment for cellular communication, biomolecular transport, and defense against antibiotics and phage invasion. A variety of terms has been used to describe EVs according to their origin. For example, cytoplasmic membrane vesicles (CMVs) are used to call EVs from Gram-positive bacteria, while those from Gram-negative bacteria are termed outer membrane vesicles (OMVs). Mammalian EVs are historically called exosomes or ectosomes (also referred to as microvesicles) [9,10]. In this review, we use extracellular vesicles (EVs) to collectively refer to all secreted membrane vesicles to reflect their common destination (i.e., in the extracellular environment). Regardless of their origin, EVs are lipid bilayer-enclosed structures displaying glycoproteins and glycolipids on their surface while encapsulating biomolecules cargoes such as cytosolic proteins, organelle, RNA, and DNA within. Of note, while a repertoire of biomolecules on the surface of and within EVs generally follow those of parental cells, certain biomolecules are selectively enriched or depleted depending on the state of the parental cell [11,12,13]. In the past decades, EVs have emerged as attractive molecular cargo to encapsulate and deliver payloads of interest, including small-molecule drugs, therapeutic proteins, and gene-editing components. In addition, EVs’ surfaces can be functionalized with exogenous molecules, such as glycans, glycoproteins, or antibodies, to enhance cellular targeting. In this review, we will focus our discussion on the use of EVs to deliver carbohydrate-based therapeutics and vaccines. We refer to other exemplary reviews for broader biomedical applications of EVs [14,15,16].

### 1.1. EVs and Their Biogenesis

#### 1.1.1. Biogenesis of Mammalian EVs

**Figure 1:**
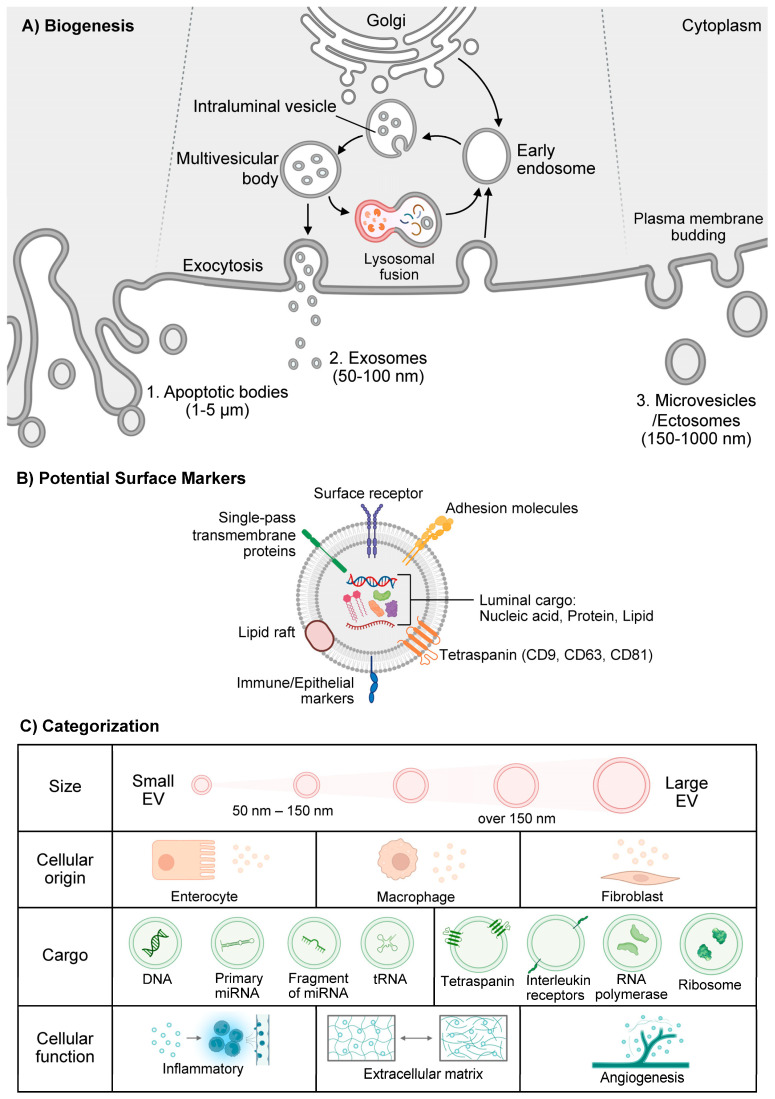
Mammalian extracellular vesicles. (**A**) Three distinct biogenesis pathways are responsible for releasing EVs from cells. These EVs differ in size, as indicated, and encapsulated cargoes depending on the biogenesis pathway. Exosomes are secreted as part of the endosomal pathway via MVBs. Exocytosis releases exosomes into the extracellular space when the MVB fuses with the plasma membrane. Microvesicles/ectosomes are released directly via outward budding of the plasma membrane. Apoptotic bodies are formed during programmed cell death. Their biogenesis involves membrane blebbing, formation of apoptopodia, and frequently includes fragmented nuclear material. (**B**) Representative of surface proteins used as markers to identify EVs and their origins. (**C**) Examples of criteria used to categorize EVs from mammalian cells. Figure was created with BioRender.

All mammalian cells produce EVs and these naturally occurring nanovesicles are found in all biological fluids including blood, synovial fluid, urine, and saliva [17]. In humans, EVs are produced from three distinct biogenesis pathways, producing subtypes of EVs including microvesicles, exosomes, and apoptotic bodies from programmed cell death or apoptosis (ApoEVs) [17,18] (Figure 1).

Exosomes are small EVs with 50–150 nm in diameter (Figure 1). They are released as part of the endosomal pathway [17]. Exosomes are formed through inward budding of the late endosomal membrane to create intraluminal vesicles (ILVs) within multivesicular bodies (MVBs) [17,19]. When the MVB fuses with the plasma membrane, these ILVs are released as exosomes into the extracellular space [17,19]. The biogenesis of exosomes involves several key mechanisms with the endosomal sorting complex required for the transport (ESCRT) dependent pathway representing a major mechanism that sorts ubiquitinated cargo into ILVs within MVBs [17,19]. Independently from ESCRT, exosome formation can also occur via lipid-mediated mechanisms where ceramide lipids induce membrane curvature [17,19]. Additionally, the scaffold protein syntenin interacts with syndecans and the ESCRT accessory protein ALIX to promote exosome formation [19].

Exosomes serve as one of the critical components for intercellular and intra-organ communication [20]. Exosomes carry biomolecular signals from one type of cell or tissue to another by gaining access to the interstitial space and circulation, where they can exert local paracrine or distal systemic effects [20]. This is a result of exosomes’ unique ability to cross important biological barriers including tissue penetration, diffusion into the blood, and even crossing the blood–brain barrier (BBB) [21]. These capabilities allow exosomes to deliver their cargo to previously hard-to-reach areas of the body, making them promising vehicles for drug delivery to the brain and other protected tissues. Exosome uptake is often mediated by specific proteins and biomolecules present on the exosome surface [22]. For example, exosomes can selectively transfer to specific organs like the kidney or be preferentially taken up by endothelial cells or pancreatic cells and such selectivity is based on exosomal tetraspanin-associated receptors that bind to ligands on the surface of the target cells [23,24]. The tissue-specific targeting capability and ability to cross important biological barriers makes exosomes valuable for targeted drug delivery applications.

Additionally, exosomes also play important roles in metabolic processes. Insulin sensitivity can be modulated by exosomes. For example, exosomes from obese adipose tissue can inhibit insulin sensitivity [25,26,27,28], while those from lean adipose tissue macrophages can enhance insulin sensitivity [20,29]. Additionally, circulating exosomes from obese subjects can produce glucose intolerance when administered to lean mice [30,31,32]. Interestingly, adipocytes can release lipid-filled exosomes that are found to be able to induce differentiation of immune cell precursors into adipose tissue macrophage, highlighting the role of EVs in proper cell–cell communication [33].

Microvesicles, also known as ectosomes, are larger EVs with 150–1000 nm in diameter (Figure 1). They are formed through direct outward budding of the plasma membrane [17,19]. Their biogenesis involves cytoskeleton rearrangement, lipid redistribution, and small GTPases. The process of cytoskeleton rearrangement involves phospholipid redistribution and contraction of the actin cytoskeleton to allow vesicle pinching and detachment [17,19], while lipid redistribution confers changes in membrane lipid composition, including exposure of phosphatidylserine on the outer leaflet that facilitates microvesicle formation [19]. Additionally, small GTPases such as ARF6 and RhoA proteins have been shown to regulate microvesicle release [19].

Microvesicles function as intercellular communication agents [34,35]. They can deliver multiplexed information to surrounding tissues and even throughout the body [35]. Without the need for membrane fusion, microvesicles can bind to receptors on the surface of target cells and induce signaling [36]. Then, upon fusion with target cell membranes, microvesicles release their luminal contents into the cytosol, activating various signaling pathways [37,38,39,40,41]. This transfer of cargo can induce significant changes in the physiology of target cells [34]. For example, the transfer of nucleic acids such as miRNAs can alter gene expression [42,43,44,45,46,47,48], which can alter cell state, cell survival, and other essential cellular processes [42,43]. In addition, microvesicles derived from stem or progenitor cells play roles in organ repair and protection against diseases [49]. In normal physiology, microvesicles are involved in maintaining stem cell plasticity [50], facilitating communication between mother and fetus during pregnancy [51], and are involved in the blood clotting process [52].

Apoptotic bodies are the largest EVs with 1–5 µm in diameter (Figure 1). They are produced during programmed cell death [17,19] and their biogenesis involves membrane blebbing and formation of apoptopodia. Apoptotic bodies often contain fragmented nuclear materials [19].

A primary function of apoptotic bodies is to facilitate an efficient removal of cellular debris following apoptosis event. At the final phase of apoptotic death, cells divide into apoptotic bodies, which are then phagocytosed by macrophages, parenchymal cells, or neoplastic cells [53,54]. This process is crucial for maintaining tissue homeostasis and preventing inflammatory responses typically associated with cell death [55,56,57]. Apoptotic bodies can also serve as vehicles for the horizontal transfer of genetic material and signaling molecules, carrying bioactive cargoes including organelles, nuclear fragments, proteins, and nucleic acids from dying cells to recipient cells [18,58,59,60].

Apoptotic bodies also play significant roles in modulating immune responses. They can promote anti-inflammatory responses in macrophages [61,62,63]. Conversely, they can generate sterile inflammation in some contexts, for example, through the carriage of IL-1α from endothelial cells [64]. In addition, apoptotic bodies from infected cells can trigger antimicrobial immunity [65]. Furthermore, they can initiate antitumor immunity by transferring tumor antigens to antigen-presenting cells like dendritic cells, activating adaptive T cell responses through cross-presentation [66,67,68,69,70].

Extracellular vesicles derived from apoptotic bodies of the mature endothelial cells can promote proliferation and differentiation of endothelial progenitor cells, potentially contributing to vascular repair [59]. Using zebrafish epithelia as a model, caspase 3-induced apoptotic bodies carrying Wnt8a from dying epithelial stem cells promoted proliferation of neighboring cells [71]. Also, MSCs-derived apoptotic bodies can promote angiogenesis and cardiac functional recovery after myocardial infarction [72]. More recently, it was shown that cardiomyocyte-derived apoptotic bodies improve heart systolic function in heart failure models [73]. Bone marrow MSCs-derived apoptotic bodies can also enhance migration and proliferation of fibroblasts as well as accelerate skin wound healing by inducing the polarization of macrophages to the M2 phenotype [74]. Bone remodeling is also influenced by apoptotic bodies as apoptotic bodies derived from osteoclasts participating in bone remodeling through RANKL reverse signaling in osteoblasts [75].

#### 1.1.2. Biogenesis of Bacterial EVs

**Figure 2:**
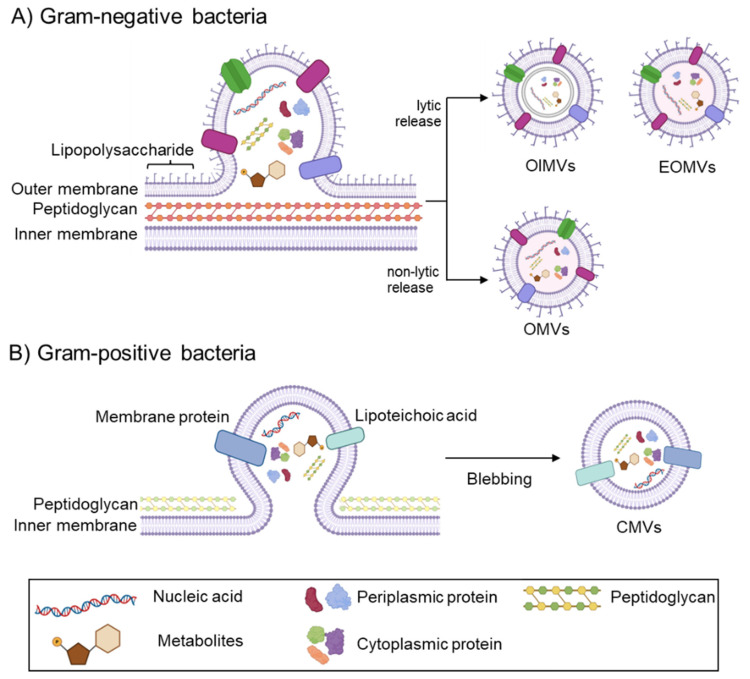
Bacterial EVs biogenesis. (**A**) In Gram-negative bacteria, non-lytic release due to imbalanced cell wall biosynthesis or intercalation from outside hydrophobic molecules leads to a formation of the outer membrane vesicles (OMVs). Lytic processes typically induced by bacteriophage infection generate outer–inner membrane vesicles (OIMVs) and explosive outer membrane vesicles (EOMVs). (**B**) In Gram-positive bacteria, CMV formation is triggered by the disruption of the cell wall peptidoglycan, which can result from autolysin or endolysin activity, antibiotic treatment, or phage infection, the latter being induced by phage-derived endolysin. Figure was created with BioRender.

Bacterial EVs are non-replicative membranous structures released into the extracellular environment and vary in origin, size, composition, and function [76,77,78]. Both Gram-negative and Gram-positive bacteria can release extracellular vesicles (EVs) without energy consumption [79,80]. Bacterial extracellular vesicles (BEVs) are heterogeneous in sizes, ranging from 20 to 400 nm in diameter, and they play important roles in intercellular communication and signaling [81,82]. Bacterial extracellular vesicles (BEVs) are categorized into four subtypes: outer-membrane vesicles (OMVs), explosive outer-membrane vesicles (EOMVs), outer–inner membrane vesicles (OIMVs), and cytoplasmic membrane vesicles (CMVs) (Figure 2). OMVs, EOMVs, and OIMVs are produced by Gram-negative bacteria, while CMVs are formed by Gram-positive bacteria [81,82]. Due to differences in the cell wall structure between Gram-negative and Gram-positive bacteria, the biogenesis and composition of bacterial extracellular vesicles are inherently distinct (Figure 2).

In Gram-negative bacteria, vesiculation is stimulated by outer membrane curvature-inducing stressors such as disruptions in the linkage between the outer membrane and peptidoglycan [83,84] or the accumulation of phospholipids in the outer leaflet of the outer membrane [85] (Figure 2A). OMVs are formed by the non-lytic release of the outer membrane, while OIMVs and EOMVs are generated through the lytic release of the outer membrane [81,82,85]. OMVs and OIMVs share common components, including lipopolysaccharides (LPSs), membrane lipids, peptidoglycan (PG), outer membrane proteins (OMPs), periplasmic proteins, and metabolites; however, OIMVs are typically enriched with cytoplasmic components, such as nucleic acids, and inner membrane (IM) elements when compared to OMVs [86,87,88,89,90]. Distinctively, EOMVs represent multiple vesicles within a larger vesicle or irregularly shaped inner vesicle, high levels of fragmented genomic DNA, enrichment in prophage-encoded mRNAs, and frequent association with phages, both on the surface and within the vesicles [81]. In Gram-positive bacteria, EV formation is triggered by the disruption of the cell wall peptidoglycan, which occurs due to autolysin or endolysin activity, as well as through antibiotic treatment [91,92] (Figure 2B). In this process, the cytoplasmic membrane blebs outward through weakened areas in the cell wall and eventually pinches off to form cytoplasmic membrane vesicles carrying nucleic acids, membrane and cytoplasmic proteins, membrane lipids, lipoteichoic acids (LTAs), and other metabolites [86,93,94,95].

Natural bacterial EVs play crucial roles in bacterial survival, cell communication, infection, and bacterium–bacterium as well as bacterium–host interactions. For instance, bacterial EVs support survival by acting as decoys to mitigate toxic compounds such as toluene [96], polymyxin B [97], and colistin [98], and to neutralize environmental agents such as antimicrobial peptides that target the outer membrane [99]. Bacterial EVs also facilitate a release of the attacking phages [100,101] or stress-induced products like misfolded periplasmic proteins [102]. Further, studies have shown roles of EVs in promoting the formation of bacterial biofilms and communities [103]. Additionally, bacterial EVs can deliver information to target cells via effector molecules through mechanisms such as membrane fusion, endocytosis [104,105,106], or targeted delivery mediated by the external surface of bacterial EVs [13,107].

Bacterial EVs possess unique properties, including the ability to display pathogen-associated molecular patterns (PAMPs), and to modulate host immune system. These features enable bacterial EVs for use as efficient vehicles for drug and vaccine delivery. This is particularly useful in immunotherapy as the bacterial antigens can enhance immune response-targeting tumor cells. Additionally, bacterial EVs contain many of the same immunogenic components as the pathogens but lack genetic material, making them a safe and effective platform for vaccine development [108]. Consequently, bacterial EVs hold significant promise for applications in biotherapeutics, including tumor immunotherapy, tumor vaccine development, infectious disease vaccines, and targeted drug delivery.

### 1.2. Glyco-Biomedicine

#### 1.2.1. Biogenesis and Biological Significance of Glycan

When there is life, there is glycan. Glycans are complex carbohydrates found across all domains of life. Glycan is considered one of the major ‘languages’ in biology complementing nucleic acids and proteins. Glycan is attached to other biomolecules (i.e., proteins, lipids, and more recently RNA [109]) via an enzymatic reaction called glycosylation to produce glycoproteins, glycolipids, and proteoglycans (i.e., glycoconjugates). Glycosylation is a highly complex and dynamic process and is essential for numerous cellular and organismal functions. Glycans play crucial roles in protein folding and functions, cell–cell communication, developmental process, and host–pathogen interaction [110]. In humans, glycoconjugates are composed of nine basic monosaccharides linked together to form highly diverse linear and branched glycan structures. Further modifications on glycans including sulfation, phosphorylation, and acetylation are possible, and together, these give rise to a great complexity of glycans [111].

Glycan is not a primary gene product (i.e., non-template synthesis). In eukaryotes, glycan is a result of complex metabolic networks driven by hundreds of glycosylation enzymes, primarily residing within the endoplasmic reticulum (ER) and Golgi body. The repertoire of glycans in a given cell or organism is called glycome. Human glycome is orchestrated by at least 200 glycosylation enzymes (174 glycosyltransferases and 35 sulfotransferases), organized into 14 unique glycosylation pathways [112]. Almost all proteins passing through secretory pathways are glycosylated, with N-linked and GalNAc-type O-linked glycans being the major types. Many nucleoproteins are also commonly glycosylated via an O-GlcNAcylation pathway [113]. These glycans serve numerous crucial biological functions. For example, O-glycans on the linker region of the low density lipoprotein receptor (LDLR) driven by the GalNAcT11 enzyme affect ligand binding and thus are critical for cholesterol homeostasis [114,115]. Not surprising, aberrant change in glycosylation enzymes/pathways, and thus resulting glycans, can lead to malignant or disease transformation. One illustrative example is a truncation of O-glycan on the hinge region of IgA. This results in humoral response against Tn(GalNAc-ɑ)-IgA epitope, forming an immune complex that blocks glomerulus and leads to IgA nephropathy [116]. Thus, understanding changes in glycosylation is critical to gain insight into underlying mechanisms in disease progression and to deduce better, targeted interventions. Truncation of glycan on the mucin 1 (MUC1) protein found on the membrane of almost all human cells is one of the major hallmarks in cancer progression [117]. Truncated O-glycan (i.e., Tn or GalNAc-ɑ) on MUC1 is found to be immunodominant epitope and high affinity antibodies against this epitope has been raised [118], with several clinical trials deploying the antibody as antibody-based therapy and/or CAR-T therapy are in progress [119].

In prokaryotes, glycans are commonly found as one of the major structural components on cell walls. Both Gram-positive and Gram-negative bacteria can produce capsular polysaccharides (CPSs) while Gram-negative bacteria also express lipopolysaccharides (LPSs) [120]. Importantly, prokaryotes employ a much greater diversity of monosaccharide subunits (over 100 different monosaccharides) [121] and thus produce far greater diverse glycan epitopes than those in humans. Such diversity is a basis for the hypothesis that glycan chains on the surface of bacteria are its signature molecule that can be exploited as a target for vaccination [122]. Indeed, several licensed vaccines based on bacterial polysaccharides are commonly used and we will discuss these in a later section. In Gram-negative bacteria, the outer membrane polysaccharide, also termed O-antigen, is first assembled as a monomer unit (3–5 monosaccharides) on the undecaprenyl-diphosphate lipid molecule residing on the cytosolic face of the inner membrane. The lipid-linked glycans are then flipped to the periplasmic face on the inner membrane and the polymerase enzyme then assembles these monomers into higher molecular weight glycan forms of the outer surface glycan. Finally, the polysaccharide is ligated to a lipid A molecule before being shuttled to the surface [123]. It is important to note that the structural heterogeneity of the O-antigen is vast, even within the same species, and can occur in response to environmental changes as well as stages in symbiosis and pathogenesis. Thus, care should be given when selecting species-specific O-antigen as targets for vaccine development.

Finally, it was previously thought that Gram-negative bacteria lack protein glycosylation machinery but this dogma was challenged with a discovery of bona fide protein glycosylation pathways in bacteria species including N-linked protein glycosylation in the human gastrointestinal pathogen *Campylobacter jejuni* [124] and O-linked protein glycosylation in the Neisseria spp. [125,126]. Both N- and O-glycans can be assembled and glycosylated either by en bloc transfer or stepwise mechanism. In the en bloc transfer mode, glycan is first assembled on lipid carriers before being transferred onto target proteins by oligosaccharyltransferase (OST) enzymes [127]. This mode of glycosylation is described for biosynthesis of the N-glycoproteins in *Campylobacter* and *Helicobacter* spp. [124,128], of the O-glycoproteins in Gram-negative bacteria of *Neisseria*, *Acinetobacter*, and *Francisella* spp. [125,129,130], as well as of the O-mannosylated proteins in the Gram-positive actinomycetes [131,132]. Protein glycosylation via stepwise reaction is catalyzed by glycosyltransferases (GTs) which transfer monosaccharides from nucleotide-activated sugar directly onto acceptor proteins. This glycosylation mode is identified for N-glycoprotein biosynthesis in *H. influenzae* [133] as well as the O-glycoprotein of bacterial flagellins in *Campylobacter* and *Helicobacter* spp. [134,135]. It has now been established that N- and O-glycoproteins produced in these bacteria play key roles in their pathogenicity including steps for adhesion, immune evasion, and host colonization [127].

#### 1.2.2. Production of Glycoprotein Therapeutics and Glycoconjugate Vaccines

The first therapeutic protein drug was a mixture of polyclonal antibodies called serum therapy used to treat diphtheria in the late 19th century [136]. Today, highly purified monoclonal antibodies (mAbs) are dominating the biopharmaceutical industry, accounting for more than half of the total products with market value reported over USD 200 billion [137]. Antibodies and a plethora of other therapeutic proteins including human erythropoietin (EPO) and granulocyte colony-stimulating factor (G-CSF) are glycoproteins and their glycans are important for the biological functions and pharmacokinetics/dynamics. Therefore, considerable efforts have been dedicated to the development of a platform for producing these therapeutic proteins with controllable and reproducible glycan profiles. Here, we will focus our discussion on glycoprotein therapeutics and vaccines produced from cell-based and cell-free systems that can be used in drug formulation with EVs. We would also like to refer to other reviews for total chemical synthesis of glycans and glycoconjugates [138].

Cell-based production—As mAbs continue to lead global biopharmaceutical sales, the mammalian system, in particular Chinese hamster ovary (CHO) cells, remains the most commonly used expression system. CHO culture is well known for its high antibody titer production, reaching up to 8 g/L at production scale [139]. Human IgG contains conserved N-glycans at the Asn297 of the Fc hinge region with biantennary N-glycoform as the most common structure. Antibodies produced from CHO cells typically have Fc glycans similar to human ones but some low immunogenic glycoforms such as oligomannose can be found [140]. Fc glycan directly affects antibody-dependent cellular cytotoxicity (ADCC) function of the antibody and glycoengineered efforts have yielded great insight into structure–function relations of the Fc N-glycans. For example, increasing bisected N-glycoform which resulted in the depletion of core fucose could enhance ADCC of the IgG by ~100-fold [141]. The first glycoengineered, afucosylated antibody Mogamulizumab targeting CC chemokine receptor type 4 (CCR4) was approved in Japan in 2012 for treating Sézary syndrome and since then several glycoengineered antibodies have been approved for use in clinical trials [142].

Recent progress in precise gene editing (ZFN, TALEN, CRISPR-Cas9) systems have allowed the glycan biosynthesis pathway to be functionally annotated and modified. Precise gene editing tools have also permitted the creation of a large library of glycoengineered mammalian cell lines capable of producing glycoproteins with designer glycan features [143,144,145,146]. Production of glycans using glycoengineered human cells has advantages on EVs-based vaccine production as the major components on EVs are human-derived and thus limit unwanted immunogenicity. However, it is important to note that glycosylation patterns on EVs are not always consistent with the parental cells [147,148,149] and the immunogenicity of EVs should be validated on a case-by-case basis. Glycoengineering strategy can also be deployed to generate EVs with desired surface glycan outcome [150]. We will discuss this strategy in more detail in a later section.

Historically, prokaryote fermentation is used for a production of therapeutic proteins without PTMs like glycosylation as there is no bona fide glycosylation pathway in bacteria. As mentioned above, authentic protein N- and O-glycosylation pathways have been discovered in several bacterial species. Biological studies and engineering efforts have now allowed us to recapitulate these glycosylation pathways in simple model organisms including *Escherichia coli* [151], and glycoengineered *E. coli* have been proposed for use as a platform for producing therapeutic glycoproteins. While structures of bacteria glycans are significantly different from those of humans and these can be immunogenic, metabolic and pathway engineering strategies have been employed to create *E. coli* strains capable of producing authentic human N- and O-glycans [152,153]. Noteworthy, bacterial glycosylation pathways and enzymes, for example OSTs, are relatively simpler than eukaryotic counterparts. This has permitted glycosylation components to be purified for structural and functional characterization [154,155] or to be engineered to enhance or alter catalytic activities [156,157,158,159]. Of note, platforms for expression and selection of full-length IgG antibodies in *E. coli* exist [160,161,162], and we anticipate that integrating glycoengineered *E. coli* with an *E. coli*-based IgG expression platform will allow for a production of full-length antibody bearing authentic human glycans from *E. coli* cells. Finally, owing to the relaxed substrate promiscuity of *E. coli* O-antigen ligase, it has been established that glycoengineered *E. coli* can recombinantly produce a diverse array of O-antigens [163]. These O-antigens can either be transferred onto proteins for producing purified glycoconjugate [164] or displayed on the surface of *E. coli* cells and its derived EVs to produce EVs-based vaccines [165].

Cell-free production—the ability to precisely control components and conditions as well as supplementing with unnatural monosaccharide units—has made cell-free reaction another attractive avenue for production of glycans and glycoconjugates. Chemoenzymatic synthesis using purified glycosyltransferase and glycosyl hydrolase has been established and a large repertoire of these glycoenzymes has been tested [166,167,168,169]. Chemoenzymatic reactions can be deployed in a variety of formats including traditional reaction tubes, microfluidic devices, as well as fully automated systems [170]. The advantage of this approach is the ability to combine substrate proteins and glycans from various sources and to precisely control reaction conditions. However, the cost associated with purified components has been an inhibitory factor in realizing this approach for a production scale. Nonetheless, an all-purified system is highly useful for prototyping novel glycosylation reactions and pathways as well as for producing highly purified, structurally well-defined glycoproteins for structural and functional studies. Alternatively, an *E. coli*-based cell-free synthetic glycobiology system pioneered by Jewett and DeLisa groups has emerged as an attractive platform combining freedom of operation of the cell-free system with cost efficiency from the use of crude cell lysate [171]. This system leverages *E. coli* cell lysate carefully prepared to retain active transcription, protein translation, and protein glycosylation machineries for a production of glycoproteins including N-glycosylated EPO [172] and human mucin-1 with authentic human O-glycans [153] as well as glycoconjugate vaccines [173]. The scalability of *E. coli*-based cell-free systems has also been demonstrated with a 100 L reaction volume for producing GM-CSF [174]. Another advantage of the cell-free systems is the fast reaction time which is particularly useful for functional screening of novel proteins and glycosylation machineries [175], and this has been demonstrated recently for antibody discovery [176]. While *E. coli* lysate is the predominant cell-free system, mammalian lysates including those from CHO and HEK293T have gained some attractions due to their ability to support protein complex formation and incorporation of PTMs including phosphorylation and glycosylation. Production of therapeutic proteins including EPO [177] and antibodies [178] have been reported in mammalian-based cell-free systems but the ability to reproducibly obtain glycan profiles on these proteins remains a subject for further investigation.

## 2. Preparation of EVs as Nanovehicles for Drug and Vaccine Delivery

### 2.1. EVs’ Source and Isolation

All organisms exhibit the capability to produce EVs [179]. Mammalian EVs, and, in particular, those from human cells, are readily biocompatible for biomedical uses such as for drug delivery, while Gram-negative bacteria EVs have been demonstrated for their potential as vaccines. Thus, these two EVs constitute a primary focus of research for EVs-based pharmaceutical applications [179,180,181]. Over the past few decades, various methods have been investigated for a preparation of EVs for biological studies as well as for therapeutic deployments. In Table 1, we provide summary of EV isolation methods, grouping these into traditional and emerging techniques. It is critical to note that the isolation method of choice should be chosen based on downstream applications, with careful considerations given to EVs’ purity, integrity, and immunogenicity. Furthermore, isolation methods can be performed in tandem to increase the purity of the EVs’ product. For example, immunoaffinity with AsFlFFF can enable automated isolation and fractionation of EV subpopulations [182]. Finally, many isolation techniques are now being integrated into microfluidic devices for rapid, automated EV isolation [182,183].

With an increasing interest of EVs in therapeutics, more emphasis is now placed on the scalable production of EVs. Cells naturally release EVs at low abundance. As cellular release of EVs is subjected to cultivation conditions, modulating culture parameters including temperature, pH, and oxygen content have been shown to affect EVs yield [224,225,226,227,228]. Restriction of fetal bovine serum (FBS) from media was also found to increase EV secretion for certain myeloma cell lines [226], but the contradicting result was also reported for other cell lines [229]. It is noted that a considerable amount of EVs is present in the standard FBS reagents, and this must be taken into consideration when evaluating EV yield. Several physical stimulations including shear stress [230], acoustic treatment [231], magnetic force (patent WO2021086139), and irradiation [232] have been investigated for increasing EVs production. Further, a large array of chemical inducers have been tested for stimulating EVs secretion (see review [233]). Regardless of the methods, it is critical to realize that stimulants can significantly affect molecular profiles of the secreted EVs and thus each EVs batch should be carefully quality-validated in parallel to the yield quantification. Currently, there is no standardized protocol for EV yield quantification. A meta-analysis of ~260 EVs-related studies found the total protein amount (via bicinchoninic acid (BCA) assay) and number of particles (via NTA) per million cells to be the most common methods for yield quantitation [234]. Among these, commercial reagents (e.g., Total Exosome Isolation Reagent) provided the highest yield at ~4.0 ug total protein per million cells, while ultracentrifugation with gradient separation yielded the lowest EVs amount at ~2.2 ug/million cells. Standard ultracentrifugation, with or without washing steps, provided a similar yield at ~3.0 ug/million cells [234]. It should be noted that while total protein amount is a well-established, rapid, and robust protocol, this does not exclude signals from protein coprecipitation with EVs. Further, total protein yield does not provide quality information of the isolated EVs. Together, these highlight a need for standardized methods and protocols for assessing EVs’ yield and quality.

### 2.2. EVs’ Characterization

EVs are heterogeneous in size, origin, and molecular constituents, making their characterization a complex task [235]. In general, EVs are characterized for (i) their physical properties including particle size, density, morphology, and transport parameters, and (ii) their molecular profiles which can reveal their origins and state of the parental cells. Both traditional and omics approaches have been employed to analyze EVs, each offering unique insights into their properties and functions (Table 2). Of particular interest, the omics approach aims at revealing total composition and quantitation of biomolecules within EVs that are increasingly popular, and we believe such an approach will greatly enhance our understanding of EVs’ biology as well as pave a way for making full synthetic EVs with fine-tunable properties.

### 2.3. Glycoengineering of EVs and Their Payload

Glycoengineering of EVs’ parental cells as well as the EVs’ payload have been proposed to enhance target specificity. Shimoda et al. hypothesized that cell–EV interactions could be modulated by glycoengineered EVs [307]. By profiling glycans on natural and glycoengineered EVs using lectin microarray, the authors demonstrated that surface glycans of the EV can be chemoenzymatically modified using glycosyltransferases or glycosyl hydrolases and this strategy can be leveraged to fine-tune EV cellular uptake [307]. Furthermore, EVs’ glycan can be modified to improve their tracking and biodistribution, to boost their immunogenicity for cancer vaccines, and to enable better methods for their detection and isolation [308]. These improvements expand the biomedical applications of EVs in diagnosis and drug delivery.

EVs can also be glycoengineered for vaccine development. For example, incorporation of a transmembrane glycoprotein namely the G-protein of the vesicular stomatitis virus (VSV-G) into exosome-like vesicles (ELVs) was found to significantly enhance their cellular uptake and stimulates dendritic cell maturation [309]. ELVs containing ovalbumin (a model antigen) and displaying VSV-G were found to facilitate cross-presentation of ovalbumin, mediated through an endosomal acidification pathway [309]. Mice vaccinated with these ELVs showed an elevated IgG2a antibody response and a strong in vivo cytotoxic T-cell lymphocyte (CTL) response leading to an enhanced protection against challenge with ovalbumin-expressing tumor cells [309].

More recently, a layer of carbohydrate coating on cell surface, known as glycocalyx, was found to be a critical determinant for efficient DC targeting [310]. Thus, strategic modifications to the EV glycocalyx have a potential to enhance the efficacy of EVs as anti-cancer vaccines [310]. Similarly, the use of α-D-mannose for surface modification of bovine serum-derived exosomes (EXOs) was shown to facilitate interaction with mannose receptors highly expressed on DCs and this can be used to optimize the delivery of immune stimulators to DCs [311]. Additionally, incorporation of the adjuvant such as monophosphoryl lipid A (MPLA) in this system can further enhance the immune response [311]. Following intradermal administration, there was a higher retention within lymph nodes, suggesting their potential as carriers for the in vivo delivery of immune stimulators and antigens to lymph nodes [311].

While glycoengineering strategy was mainly applied to exosome EVs subtype, there exist works exploring potential use for apoptotic body and microvesicle. Apoptotic tumor cell-derived extracellular vesicles (ApoEVs) were generated for use as tumor vaccine [66]. ApoEVs was considered a rich source for tumor-specific neo-antigen and other tumor-associated antigens (TAAs) [311]. Further, ApoEVs can be incorporated with DC-targeting ligands, effectively enhancing its immunogenicity [66]. By strategically modifying the glycocalyx of the tumor cells, it is possible to induce surface expression of high-mannose glycans on both the parental cells and their ApoEVs [66]. These high-mannose glycans serve as ligands for dendritic cell-specific intercellular adhesion molecule-3-grabbing non-integrin (DC-SIGN), a C-type lectin receptor (CLR) specifically expressed on DCs [66]. DC-SIGN possesses the capacity to efficiently uptake its ligands, directing them towards both major histocompatibility complex (MHC)-I and MHC-II pathways, thereby stimulating the response of both CD8+ and CD4+ T-cells [66]. Compared to unmodified one, ApoEVs displaying DC-SIGN ligands exhibit significantly enhanced cellular uptake by DCs, leading to enhanced priming of tumor-specific CD8+ T cells [66]. This innovative approach demonstrates promise as a novel vaccination strategy to enhance the efficacy of T cell-based cancer immunotherapies [66].

It was found that MUC1, important tumor-associated glycoprotein, is present on microvesicles that undergo intracellular translocation from the endo-lysosomal/HLA-II compartment to the HLA-I processing pathway within DCs, presented by DC to MUC1-specific CD8+ T-cells that subsequently induce IFN-γ production [312]. In contrast, soluble MUC1 remains confined to the endo-lysosomal/HLA-II compartment, influenced by both the O-glycans on MUC1 and the specific mode of cellular internalization, whether receptor-mediated or independent of specific receptors [312]. This finding suggests that microvesicle-mediated transfer of tumor-associated glycoproteins to DCs represents a biologically significant mechanism in vivo, potentially influencing the nature of the elicited immune response [312]. These results have profound implications for the design of glycoprotein-based immunogens for cancer immunotherapies and vaccines [312].

## 3. The Application of EVs in Disease Treatment

### 3.1. EVs in Neurological Diseases

#### 3.1.1. Alzheimer’s Disease (AD)

**Figure 3:**
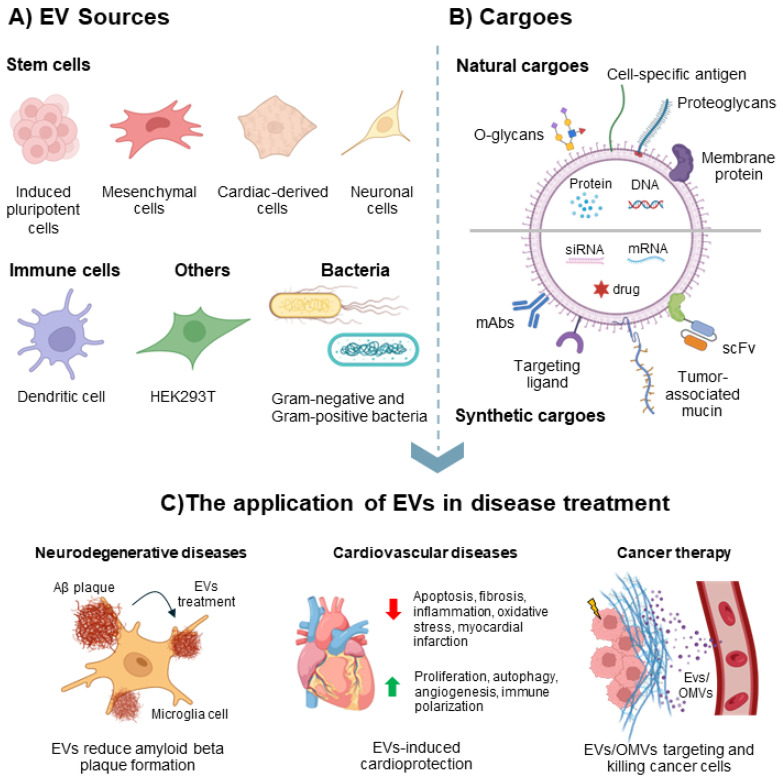
Biomedical application of EVs. (**A**) Human and bacterial cells can be used to source EVs. (**B**) EVs contains myriads of biomolecules derived from parental cells. These natural cargoes include surface-displayed N-, O-, glycosaminoglycans, glycolipids, and glycoconjugates as well as encapsulated genetic materials (DNA, mRNA, miRNA, etc.), cytosolic proteins, and metabolites. Engineering strategies including genetic, metabolic, and in vitro modifications can be deployed to load EVs with synthetic cargoes such as mAb/scFv for targeting or cancer-associated O-glycomucins domains for eliciting cancer-specific immune responses. (**C**) EVs have been proposed for use in disease treatment with illustrative examples include (i) reduction in neuritic plaques implicated for pathogenesis of Alzheimer’s disease; (ii) providing cardioprotection and/or tissue and vascular repair; and (iii) delivery of cytotoxic drugs, immune blockages, or immune adjuvants to suppress and eradicate tumors. Figure was created with BioRender.

Alzheimer’s disease (AD) is a progressive neurological disorder characterized by memory decline and cognitive impairment, primarily driven by microglia-mediated inflammation. It is the most common neurodegenerative disease and the leading cause of dementia in the elderly. AD is associated with the excessive accumulation of amyloid-β (Aβ) peptides and neurofibrillary tangles, leading to neuronal dysfunction and eventual cell loss [313,314]. EVs have emerged as a promising therapeutic approach for neurological disorders due to their high loading capacity, low toxicity, low immunogenicity, and perhaps most importantly, their ability to cross the blood–brain barrier. This unique capability positions EVs as a potential treatment strategy for AD and other neurodegenerative diseases [315,316,317]. EVs can be engineered by genetically modifying EV-producing cells to incorporate targeting or labeling moieties and can also be loaded with functional cargo, such as siRNA, for gene silencing (Figure 3A,B).

A key hallmark of Alzheimer’s disease (AD) is the abnormal production of amyloid-β (Aβ), driven by a β-site amyloid precursor protein cleaving enzyme 1 (BACE1) (Figure 3C). BACE1 plays a central role in Aβ plaque formation, and it has become a major target in AD research [318]. Alvarez-Erviti et al. engineered dendritic cells to express Lamp2b, an exosomal membrane protein, fused with the neuron-specific RVG peptide [319]. These dendritic cell-derived exosomes were then loaded with exogenous siRNA via electroporation. The researchers demonstrated that intravenously injected RVG-targeted exosomes successfully delivered GAPDH siRNA specifically to neurons, microglia, and oligodendrocytes in the brain, leading to targeted gene knockdown. Remarkably, exosome-mediated siRNA delivery achieved a significant reduction in BACE1 expression, with mRNA and protein levels decreasing by 60% and 62%, respectively, in wild-type mice—highlighting the therapeutic potential of this approach for AD. Cui et al. further demonstrated that mesenchymal stem cell (MSC)-derived exosomes functionalized with RVG exhibited strong targeting to the cortex and hippocampus. This targeted delivery significantly improved learning and memory abilities in an animal model for AD [314]. A recent study by Khan et al. highlighted the therapeutic potential of neuronal stem cell-derived exosomes (NSC-exos) for the treatment and prevention of AD [320]. NSC-exos therapy effectively reduced phosphorylated tau (p-tau) levels and Aβ formation by suppressing kinase expression and activity, as well as genes and proteins associated with AD pathology. Additionally, NSC-exos demonstrated anti-inflammatory effects by mitigating neuroinflammation.

While the use of MSC-derived EVs as a regenerative therapy for neurological disorders, including AD, is relatively recent, preclinical studies in cell and animal models have shown great progress. Currently, a clinical trial (NCT04388982) has been approved to assess the safety and efficacy of allogenic human adipose MSCs-Exos (ahaMSCs-Exos) for use in an individual with mild to moderate AD [321]. Here, eligible participants were assigned to receive intranasal administration of EVs (5, 10, or 20 μg groups) twice a week and for 12 weeks. Follow-up evaluations were conducted, and no adverse events were reported during the trial. Although there were no significant differences in amyloid or tau deposition changes across the groups, the 10-ug-dose group demonstrated a slightly smaller reduction in hippocampal volume. Intranasal administration of ahaMSCs-Exos was found to be safe and well tolerated, with a dose of at least 4 × 10^8^ particles recommended for future clinical trials.

#### 3.1.2. Parkinson’s Disease (PD)

Parkinson’s disease (PD) is a progressive neurodegenerative disease that involves the formation of Lewy bodies, which is affected by excessive accumulation of α-synuclein (α-Syn), a protein involved in synaptic function and neuronal survival [322]. Thus, reducing α-Syn levels in brain cells has been proposed as a potential strategy to mitigate PD symptoms. Cooper et al. utilized RVG-modified extracellular vesicles (MEVs) derived from murine dendritic cells to deliver α-Syn siRNA, effectively reducing α-Syn accumulation in the brain [323]. Similarly, Kojima et al. engineered HEK293T-derived EVs with enhanced targeting, cytoplasmic delivery, and RNA encapsulation capabilities using EV production booster devices [324]. The administration of therapeutic catalase mRNA via these MEVs significantly reduced neurotoxicity and neuroinflammation in a mouse model.

Collectively, the literature highlights the potential of extracellular vesicles (EVs) in mitigating key pathological features of neurological disorders, such as neuronal death and inflammation, while also alleviate functional and behavioral impairments. Their natural ability to cross the blood–brain barrier (BBB) makes them particularly suitable for neurological treatments [325]. Engineering features such as modifying EVs with a ligands-targeting receptor at the BBB are anticipated to further enhance their effectiveness for clinical applications. In the clinical translation context, studies such as trials NCT04603326 and NCT05320250 identified EVs as potential biomarkers for early onset of Parkinson’s disease (PD). However, it is noted that, to date, there is no clinical trials for a use of EVs as delivery vehicle for the treatment of PD.

### 3.2. EVs in Cardiovascular Diseases

Cardiovascular diseases (CVDs) are the leading cause of mortality worldwide [326]. Globally, CVDs are responsible for approximately 17.3 million deaths annually, with projections exceeding 23.6 million by 2030 [327]. Over the past decade, while conventional treatments such as pharmacotherapy and surgical interventions have helped alleviate CVD symptoms and reduce mortality rates [328,329], effective clinical strategies for myocardial repair following myocardial infarction (MI) or for preventing heart failure progression remain lacking [330]. Although medications are less invasive, they can lead to organ damages and other severe side effects [331]. Similarly, despite its effectiveness, cardiac surgery is often constrained by complex procedures and post-operative complications [332]. Given the poor prognosis associated with CVDs, there is an urgent need for innovative therapeutic approaches. EVs have demonstrated their applications in CVD therapy due to their ability to withstand the extracellular environment, traverse biological barriers, and deliver bioactive molecular cargo to target cells, with their biological function varying based on donor cell state and microenvironmental conditions [15,333,334,335]. EVs containing biological cargoes, including nucleic acids and proteins regulate multiple functions in target cells, including maintaining cardiovascular balance and health, inducing pathological changes in CVDs. Previous studies demonstrated that mesenchymal stem cell-derived EVs from different origins such as bone marrow, adipose tissues, the umbilical cord, and heart contain the functional components of miRNA-19, miRNA-21, and miRNA-210. These miRNAs effectively inhibit cardiomyocyte, reduce cardiac fibrosis, promote angiogenesis, stabilize mitochondrial membrane potential, and thus lead to restoration cardiac function in vivo and in vitro models [336,337,338,339,340]. Induced pluripotent stem cells-derived EVs (iPSCs-EVs) have been extensively studied for use in CVD therapy. The iPSC-EVs also contain functional miRNA components [341,342], including miR-19, miR-20, miR-126, miR-130, and miR-17, and these have been demonstrated to exert effects on promoting angiogenesis and adjusting hypoxia as well as oxidative stress [343,344].

EVs derived from embryonic stem cells (ESCs) have demonstrated significant potential in improving cardiac function in infarcted hearts. Their therapeutic effects are primarily attributed to enhanced neovascularization, increased cardiomyocyte survival and proliferation, and reduced cardiac fibrosis. The beneficial impact of ESC-derived EVs is linked to the delivery of miR-294 from ESCs to cardiac progenitor cells (CPCs), which promotes cell survival, facilitates cell cycle progression, and stimulates proliferation [345]. Additionally, research has indicated that human CD34+ endothelial progenitor cells (EPCs) hold promise for cardiovascular disease therapy [346,347] by promoting proangiogenic paracrine activity in ischemic limb tissues [346]. EPC-derived EVs (EPC-EVs) further support therapeutic angiogenesis, contributing to the formation of new blood vessels and improving left ventricular function in myocardial infarction (MI) patients [346]. Moreover, EPC-EVs facilitate vascular regeneration by promoting the transition of fibroblasts into endothelial cells [346]. Furthermore, exosomes derived from dendritic cells (DCs) have been shown to activate endothelial cells (ECs) through the TNF-α and NF-κB signaling pathways, particularly in human umbilical vein endothelial cells [347].

Overall, stem cell-derived EVs have shown significant potentials in enhancing cardiac functions (Figure 3C). These EVs carry bioactive cargoes that play crucial roles in cardiovascular disease (CVD) therapy, exerting multiple therapeutic effects, including apoptosis inhibition, oxidative stress reduction, fibrosis attenuation, autophagy regulation, inflammatory response suppression, angiogenesis promotion, and mitochondrial membrane potential stabilization. Various types of stem cell-derived EVs, such as those from mesenchymal stem cells (MSC-EVs), cardiac-derived cells (CDC-EVs), induced pluripotent stem cells (iPSC-EVs), and dendritic cells (DC-EVs), have been extensively studied for their applications in CVD treatment. A recent clinical trial (NCT05774509) evaluated safety and efficacy of three intravenous injections of an extracellular vesicle-enriched secretome derived from cardiovascular progenitor cells in patients with severe, drug-refractory left ventricular (LV) dysfunction caused by non-ischemic dilated cardiomyopathy [348]. The trial demonstrated excellent tolerance to repeated delivery, with no adverse events reported during or after the infusions. The rationale for using the intravenous route is that the infused EV-enriched secretome may reprogram endogenous immune cells, both circulating and within peripheral organs, to adopt a reparative phenotype. These EV-modified immune cells could then migrate to the heart, promoting tissue repair and reducing inflammation, a key feature of cardiac failure [348,349].

### 3.3. EVs in Cancer Treatment

Cancer is the second leading cause of death worldwide behind cardiovascular disease [350]. Traditional treatments for cancer beyond surgical resection include radiation and chemotherapy, however, these therapies can cause serious adverse side effects due to their high killing potency but low tumor selectivity [351,352]. Moreover, radiation and chemotherapy can themselves cause development of a secondary cancer [353,354]. As an alternative, more effective treatments such as targeted therapy, hormone therapy, stem cell transplantation, and immunotherapy have been developed. The combination of standard treatments with these newer therapies can significantly prolong and improve the quality of patients’ life.

Recently, mammalian and bacterial EVs have been documented as carriers for cancer therapy. EVs can be engineered to enhance their efficacy in cancer therapy by displaying targeting antibodies, such as anti-HER2, or receptors like the immune checkpoint PD-1 (Figure 3B,C). Additionally, EVs can be loaded with cytotoxic drugs for potent elimination of cancer cells or be loaded with siRNA for targeted gene silencing (Figure 3B). The engineered targeting mammalian and bacterial EVs have also been demonstrated to enhance therapeutic effects. In the context of chemotherapy, cancer treatment mainly involves cytotoxic drugs with high killing potency, but these drugs lack specific targeting and thus cause serious cytotoxic side effects, resulting in poor therapeutic effects [44]. To ameliorate the side effects of these drugs, targeted delivery based on engineered EVs could enhance the local drug concentration at tumor site while minimizing cytotoxic side effects at the healthy cells. Tian et al. utilized mouse immature dendritic cells (imDCs) to produce exosomes engineered to express the exosomal membrane protein Lamp2b fused with the αv integrin-specific iRGD peptide (CRGDKGPDC) for enhanced tumor targeting [355]. The purified exosomes were loaded with doxorubicin (Dox) via electroporation. These iRGD-modified exosomes efficiently targeted αv integrin-positive MDA-MB-231 breast cancer cells to deliver Dox. In vivo, intravenously administered targeted exosomes selectively delivered Dox to tumor tissues, leading to tumor growth inhibition without significant, systematic toxicity. These findings highlight the potential of ligand-modified exosomes for targeted drug delivery in cancer therapy. Similarly, Qi et al. developed a dual-functional exosome-based superparamagnetic nanoparticle cluster as a targeted drug delivery system for cancer treatment [356]. These exosomes, loaded with Dox, demonstrated efficient targeting of hepatoma 22 subcutaneous cancer cells. Under a magnetic field, the dual-functional exosomes enhanced targeting capability and suppressed tumor growth. To further improve targeting, Li et al. isolated exosomes from A33-positive LIM1215 cells (A33-Exo) and loaded them with Dox [357]. Additionally, they coated superparamagnetic iron oxide nanoparticles (US) with A33 antibodies (A33Ab-US), hypothesizing that these antibodies would bind to A33-positive exosomes, forming A33Ab-US-Exo/Dox complexes to target A33-positive colon cancer cells. In vivo studies demonstrated that A33Ab-US-Exo/Dox exhibited excellent tumor-targeting ability, effectively inhibiting tumor growth and prolonging survival while reducing cardiotoxicity [357].

Gene therapy has also emerged as a promising alternative to chemotherapy for cancer treatment. This approach aims at correcting or compensating the abnormal gene expression in tumor cells. Gene therapy involves the delivery of nucleic acids, such as siRNAs and miRNAs, to modulate gene activity and inhibit tumor progression. Extracellular vesicles (EVs) can be used to shield their RNA cargoes from degradation, thereby maintaining their stability and bioactivity until they reach targeted cells. Bai et al. developed engineered tLyp-1-modified exosomes derived from HEK293T cells to enhance the targeted delivery of siRNA for silencing the SOX2 gene in human cancer cells [358]. These modified EVs exhibited high transfection efficiency in non-small-cell lung cancer (NSCLC) and significantly suppressed SOX2 expression in NSCLC stem cells. Similarly, Zhao et al. designed biomimetic nanoparticles composed of cationic bovine serum albumin (CBSA) conjugated with siS100A4 and coated with an exosomal membrane (CBSA/siS100A4@Exosome) to improve targeted drug delivery to the lung pre-metastatic niche [359]. These engineered exosomes provided enhanced siRNA protection against degradation and demonstrated excellent biocompatibility. In vivo studies revealed that CBSA/siS100A4@Exosome exhibited greater lung-targeting efficiency than CBSA/siS100A4@Liposome and achieved potent gene-silencing effects, significantly inhibiting the proliferation of malignant breast cancer cells. Additionally, Gujrati et al. developed bioengineered bacterial outer membrane vesicles (OMVs) derived from an *E. coli* mutant strain with reduced endotoxicity in human cells [360]. These OMVs were functionalized with a human epidermal growth factor receptor 2 (HER2)-specific affibody on their membrane to enhance tumor targeting [360]. The engineered OMVs were further loaded with small interfering RNA (siRNA) targeting kinesin spindle protein (KSP) [360]. Systemic administration of siRNA-loaded OMVs resulted in targeted gene silencing and induced significant tumor regression in an animal model [360].

Immunotherapy is another promising strategy in cancer treatment, offering targeted approaches to enhance therapeutic efficacy. EVs have been explored as functional tools in tumor immunotherapy to improve treatment outcomes. One of the most advanced immunotherapies, chimeric antigen receptor (CAR) T cell therapy, involves genetically modifying individual patient’s T cells to express CARs that specifically recognize tumor-associated antigens [361]. While CAR-T therapy has demonstrated rapid and durable clinical responses, it is also linked to distinct acute toxicities and remains susceptible to immunosuppressive mechanisms. To address these challenges, Fu et al. developed EVs derived from CAR-T cells, which carry CARs on their surface [362]. These CAR-containing exosomes exhibited high levels of cytotoxic molecules and effectively inhibited tumor growth [362]. Notably, in a preclinical in vivo model of cytokine release syndrome, CAR exosomes demonstrated a superior safety profile compared to conventional CAR-T therapy. Shi et al. engineered exosomes to display both anti-human CD3 and anti-human HER2 antibodies on their surface, creating SMART-Exos capable of simultaneously targeting T-cell CD3 and breast cancer-associated HER2 receptors [363]. By redirecting and activating cytotoxic T cells against HER2-positive breast cancer cells, these SMART-Exos exhibited strong and highly specific antitumor activity in both in vitro and in vivo models. Additionally, Li et al. developed outer membrane vesicles (OMVs) modified to express the ectodomain of programmed death 1 (PD1), an immune checkpoint molecule [364]. This genetic modification preserved the immune-stimulating properties of OMVs while enabling them to bind PD-L1 on tumor cells, promoting its internalization and degradation. By blocking the PD1/PD-L1 immune inhibitory axis, these engineered OMVs enhanced T cell activation and infiltration into tumor tissues [364]. Compared to both unmodified OMVs and conventional PD-L1 antibodies, the engineered OMVs demonstrated a more pronounced effect in suppressing tumor growth through a combination of immune activation and checkpoint inhibition.

Beyond targeted tumor therapy, EVs, particularly OMVs, can be deployed as a tumor vaccine due to their ability to strongly activate the innate immune system (i.e., immunoadjuvants). OMVs can be loaded with target antigens either into the lumen or on their surface [365,366]. OMVs can be designed to bind to low antigenic tumor cell membranes, effectively increasing the antitumor innate immune response. Grandi et al. identified the overexpression of the cadherin FAT1 in various tumor cell lines and selected it as an antigen to elicit an antitumor immune response [367]. They engineered OMVs with FAT1 epitope as colorectal cancer vaccine, with the goal of stimulating the production of anti-FAT1 antibodies [367]. Immunization of BALB/c and C57bl6 mice with engineered OMVs elicited anti-FAT1 antibodies and partially protected mice from the challenge against CT26 and EGFRvIII-B16F10 cell lines [367].

Tumor heterogeneity leads to significant genetic and phenotypic variations among tumor cells, resulting in diverse tumor antigens that can vary considerably between patients. This complexity makes it challenging to develop a universal OMV-based tumor vaccine carrying a single antigen that would be effective for all individuals. Cheng et al. introduced a flexible OMV-based vaccine platform known as the ClyA catcher (CC)-OMVs [368]. Their study demonstrated that OMVs displaying the TRP2_180−188_ antigen effectively suppressed B16F10 melanoma lung metastasis. Overall, modifying OMVs can enhance their immunostimulatory properties, particularly when functionalized with tumor antigens, enabling them to elicit strong antitumor immune responses and inhibit tumor growth and metastasis. To this end, we noted several ongoing clinical trials investigating the potential use of EVs for cancer drug delivery. For instance, HEK293-derived EVs are explored in trials for delivering antitumor STimulator of InterferoN Genes (STING) agonists (NCT04592484—Phase I/II) or STAT6-targeting antisense oligonucleotides (ASOs) (NCT05375604—Phase I) [369,370]. Another Phase I clinical trial (NCT03608631) investigated the optimal dose and side effects of mesenchymal stromal cell-derived exosomes loaded with KrasG12D siRNA for treating metastatic pancreatic cancer patients with KrasG12D mutation [371]. A Phase II trial (NCT01854866) administered patients with malignant ascites or pleural effusion with chemotherapeutic drugs encapsulated in tumor cell-derived microparticles [372]. The results suggested that tumor cell-derived microparticles could be a promising approach for managing malignant ascites and pleural effusion. Finally, a Phase II clinical trial (NCT01159288) combined methoxycyclophosphamide (mCTX) treatment with administration of dendritic cell exosomes (Dex) containing tumor antigens [373]. The results demonstrated that Dex enhanced progression free survival in patients with advanced non-small-cell lung cancer (NSCLC) by strengthening the antitumor immune response mediated by natural killer (NK) cells.

### 3.4. Infectious Disease

Vaccines have been regarded as one of the most cost-effective interventions to prevent morbidity and mortality from infectious diseases. Over the past five decades, vaccines against 14 common pathogens have saved an estimated 154 million lives globally, equivalent to six lives every minute [374]. By controlling, and in some cases eradicating, many devastating viral and bacterial infections, vaccines have made an unparalleled contribution to public health [375,376]. Notwithstanding, many administered vaccines have significant limitations in their efficacy and/or wider adoption. One of the most common vaccination strategies use live or attenuated pathogens to elicit an immune response, but this has significant risk as pathogenic replicative material is being introduced into the body, which especially poses conflicts to those with compromised immune systems [377,378]. As mentioned earlier, carbohydrate epitopes on the surface of bacterial pathogens (i.e., CPS and LPS) are unique to many bacterial species and these can be exploited to elicit effective immune recognition without the use of whole pathogens. However, carbohydrate molecules typically stimulate only T cell-independent responses [379,380,381], characterized by a lack of IgM-to-IgG class switching [382] and by an inability to induce secondary antibody responses after recall immunization as well as the absence of sustained T-cell memory [383].

To address these challenges, glycoconjugate vaccines have emerged as a highly effective strategy. By linking a carbohydrate epitope to immunogenic protein carriers such as CRM197 or tetanus toxoids, glycoconjugate vaccines enhance the immunogenicity of carbohydrates and elicit robust, carbohydrate-specific immunological memory [163]. Glycoconjugates have proven to be highly efficacious and safe strategy in preventing infectious diseases caused by virulent pathogens, including *Haemophilus influenzae* type b (Hib) [384], *Streptococcus pneumoniae* (23 serotypes) [385], *Neisseria meningitidis* (A, C, W135 and Y) [386], and *Salmonella* Typhi [387,388,389].

OMVs are non-replicating, immunogenic mimics of their parental bacteria, containing pathogen-associated molecular patterns (PAMPs) such as lipoproteins, lipopolysaccharides, nucleic acids, and peptidoglycans which endow OMVs with intrinsic immunostimulatory properties, effectively triggering both innate and adaptive immune responses [390,391]. Due to the high immunostimulatory ability of OMVs, antigenic glycan can be decorated on the surface of OMVs to overcome inherent low immunogenicity. Moreover, the particle size of outer membrane vesicles (OMVs) enhances their uptake by antigen-presenting cells (APCs), facilitating antigen presentation to cognate T cells. Additionally, OMVs are captured by follicular dendritic cells (FDCs), which activate antigen-specific B cells and stimulate the adaptive immune response. Due to these advantages, OMVs have increasingly been recognized in recent years as a versatile platform for vaccine development [392,393,394]. OMVs produced by Gram-negative bacteria have been utilized as delivery systems for polysaccharides and recombinant proteins. For instance, a licensed HibOMPC conjugate vaccine has demonstrated efficacy in inducing robust antibody responses in animals [395] and triggering cytokine-mediated immune responses via engagement of TLR2 [396]. Similarly, the conjugation of *Haemophilus influenzae* type b (Hib) polysaccharides to OMVs derived from *Bordetella pertussis* has shown promise as a viable approach to induce immune responses against both pertussis and Hib infections.

While using authentic OMVs from bacteria could perhaps elicit strong immune responses towards targeted pathogens, there are considerable risks associated with large-scale culturing of pathogens as well as the possibility of live bacteria contaminations in the vaccine products. As an alternative, OMVs derived from *Escherichia coli* can be engineered to produce Generalized Modules for Membrane Antigens (GMMAs), which display glycan antigens or recombinant proteins. GMMAs, originating from Gram-negative bacteria and naturally presenting O-polysaccharide chains on their surface, have been proposed as potent vaccine candidates. They serve as carriers for chemically linked polysaccharides, enabling directed conjugation to either lipopolysaccharides (LPS)/lipooligosaccharides (LOSs) or surface-exposed proteins on the vesicles [397]. This versatility has enabled the successful covalent attachment of structurally diverse polysaccharides from various pathogens—including *Neisseria meningitidis* serogroups A and C, *Haemophilus influenzae* type b, *Streptococcus* group A carbohydrate, and *Salmonella Typhi* Vi polysaccharides—eliciting strong anti-polysaccharide responses in animal models [398,399,400].

**Figure 4:**
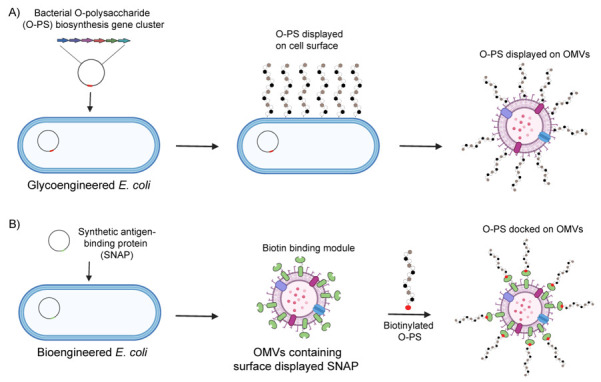
Engineering strategy for production of *E. coli*-derived OMV-based vaccine. (**A**) Recombinant expression of bacterial O-polysaccharide (O-PS) biosynthesis gene cluster in glycoengineered *E. coli* cells allows for cell-surface display of O-PS epitopes. OMVs derived from glycoengineered *E. coli* (GlycOMVs) retain O-PS on their surface. (**B**) *E. coli* cells are engineered to express and display synthetic antigen binding protein (SNAP) module on their OMVs. SNAP contains biotin binding module and thus allows for capturing of any biotinylated biomolecules, for example, biotinylated O-PS. The SNAP OMVs is a modular platform for assembling of antigen-displayed OMVs for application as vaccines. Figure was created with BioRender.

Beyond chemical conjugation, GMMAs and OMVs can be engineered to express heterologous glycans, producing glycoengineered OMVs (glycOMVs) with enhanced functionality [401] (Figure 4A). *Escherichia coli* strains lacking polymeric O-polysaccharides have been genetically engineered to incorporate operons responsible for the biosynthesis of heterologous polysaccharides into the *wbbL* gene, while preserving lipid A-core production as an acceptor. Using this approach, the heterologous glycan structure is synthesized on the cytoplasmic side of the inner membrane and assembled onto the native undecaprenyl pyrophosphate (Und-PP) carrier. The glycan is then translocated to the periplasmic side by the endogenous flippase Wzx. Once in the periplasm, the assembled polysaccharide is transferred en bloc to the lipid A-core structure by the native O-antigen ligase, WaaL [394]. Alternatively, engineered glycans can be assembled directly on the cytoplasmic side of the inner membrane, one residue at a time, starting from the terminal sugars of a truncated lipid A-core. The completed glycan is then flipped to the periplasm. Chen et al. developed a series of glycoconjugate vaccines by coordinating recombinant O-polysaccharide (O-PS) biosynthesis with hyper vesiculating *E. coli* strain JC8031, resulting in glycosylated outer membrane vesicles (glycOMVs) decorated with pathogen-mimetic glycotopes [163]. GlycOMVs were generated for eight different pathogenic bacteria, including the highly virulent *Francisella tularensis* subsp. *tularensis* type A strain Schu S4, producing *Ft*-glycOMVs. Immunization of BALB/c mice with glycOMVs elicited robust O-PS-specific serum IgG responses as well as vaginal and bronchoalveolar IgA antibodies. Notably, glycOMVs significantly improved survival following challenge with *F. tularensis* Schu S4 and conferred complete protection against challenges with two different *F. tularensis* subsp. *holarctica* (type B) live vaccine strains. Additionally, Price et al. successfully engineered *E. coli* OMVs to display the *Streptococcus pneumoniae* serotype 14 capsule (CPS14) [402]. These glycOMVs elicited serum IgG opsonophagocytic titers comparable to those induced by the corresponding chemical conjugates in PCV13. Furthermore, *E. coli* OMVs were glycoengineered to express a heptasaccharide from *Campylobacter jejuni*, which significantly reduced bacterial colonization in vaccinated chickens upon challenge [402].

Stevenson et al. generated pan-specific OMV-based vaccines by displaying conserved carbohydrate antigens, poly-β-(1–6)-N-acetylglucosamine or PNAG, on OMVs surface [403]. PNAG carbohydrate is common among numerous bacteria, fungal, and protozoa parasites [404]. The hypervesiculating *E. coli* JC8031 strain was engineered to express PNAG glycopolymer on its surface. The *Staphylococcus aureus* PNAG deacetylase enzyme, IcaB, was introduced into PNAG-expressing JC8031 cells to produce dPNAG-glycOMVs. Immunization with these glycOMVs elicited strong PNAG-specific antibody responses in mice, and only dPNAG-glycOMVs-induced antibodies are capable of effectively killing PNAG-producing bacteria in vitro, including *S. aureus* and *Francisella tularensis* subsp. *holarctica*. Importantly, this immune response protected mice from lethal doses of both *S. aureus* and *F. tularensis*.

Tian et al. successfully biosynthesized the *Shigella flexneri* 2a O-polysaccharide antigen in *Salmonella* and attached it to the core-lipid A structure for displaying on OMVs [405]. Purified OMVs were then utilized as vaccine vectors, eliciting robust anti-*Shigella* lipopolysaccharide (LPS) antibodies in serum, along with elevated IgA levels in vaginal secretions and bronchopulmonary lavage fluid following intranasal and intraperitoneal administration. The OMV vaccine conferred significant protection against virulent *S. flexneri* 2a infection, as demonstrated by serum bactericidal assays, opsonization assays, and pathogen challenge tests.

Moving beyond the integration of recombinant carbohydrate epitope expression into the OMV-producing *E. coli* strain, Weyant et al. introduced a strategy for versatile docking of carbohydrate antigens onto OMVs using an avidin–biotin interaction (Figure 4A). This method, termed AvidVax, enables virtually any biotinylated antigens to be displayed onto the surface of the OMVs that have been modified to include multiple copies of a biotin-binding protein, called synthetic antigen-binding protein (SNAP) [406]. The authors successfully decorated SNAP-OMVs with various biotinylated glycans and glycoconjugates including carrier protein CRM197 bearing *Francisella tularensis* SchuS4 O-antigen polysaccharide (FtO-PS). Of note, AvidVax was used for effectively elicited mouse immune response towards small antigens such as cancer-associated ganglioside GD2 glycans, which are inherently weak immunogens [407]. The versatility of SNAP-OMV is anticipated to accelerate development of novel vaccines as biotinylated antigens from various sources including cell-based and cell-free derived glycoconjugates can be quickly installed and tested on OMVs scaffold [408,409,410].

To date, several OMV-based vaccines have been successfully developed and deployed worldwide (see Table 3 for a full list of OMVs-based vaccines). Bexsero, a GSK (Siena, Italy) ’s product, is produced using OMV extracted from the *N. meningitidis* NZ98/254 strain and contains three recombinant proteins (NHBA, NadA, and fHbp). Together, this results in strong response to generate bactericidal antibodies against *N. meningitidis* [411]. A similar approach was used to formulate the New Zealand MenB vaccine (MenZB), and this was used to address a meningitis outbreak in New Zealand. Clinical trials conducted with the OMV-based MenZB vaccine demonstrated a favorable safety profile and a robust immune response following a three-dose regimen. Another OMV-based vaccine VA-MENGOC-BC, developed by the Finlay Institute in Havana, Cuba, was approved for use against MenB in 1987. This vaccine successfully reduced MenB disease incidence by 93–98% in the following 20 years, eventually resulting in MenB no longer being a public health problem in Cuba [412]. PedvaxHib is formulated by chemical coupling of the purified capsular polysaccharide polyribosylribitol phosphate (PRP) derived from *H. influenzae* type b with an outer membrane protein complex (OMPC) from OMV extracted from the B11 strain of N. meningitidis serogroup B [354] and this vaccine is now recommended for routine vaccination against *H. influenzae* type b in infants and children from 2 to 71 months old. In addition to monovalent vaccines, OMVs have been modified to present multiple antigens in their natural configuration. For example, PedvaxHib was combined with the hepatitis B surface antigen (HbsAg) to create a dual vaccine against Hib and hepatitis B, marketed as Comvax^®^ (Procomvax^®^ in the EU). Furthermore, PedvaxHib is included in the hexavalent pediatric vaccine Vaxelis^®^, which incorporates diphtheria and tetanus toxoids, *Bordetella pertussis* antigens, HbsAg, and inactivated poliovirus. Several OMV-based vaccines are currently in clinical development, including Avacc 10^®^ for COVID-19 (Phase I), iNTS-GMMA^®^ for invasive non-typhoidal *Salmonella* (Phase I), *Neisseria gonorrhoeae* (Phase I), and altSonflex1-2-3 for *Shigella* (Phase II).

## 4. Conclusions and Outlook

Extracellular vesicles (EVs) represent a new paradigm in biomedicine. Their unique properties, including nanosized structures, cargo-loading capacity, and modifiability, make them highly versatile for various biomedical applications. In the past decade, EVs have been extensively explored as drug delivery systems and vaccine platforms for treating infectious diseases, cardiovascular diseases, neurological disorders (e.g., Alzheimer’s disease), and cancer. EVs can encapsulate a range of biological cargo, such as functional mRNA and small-molecule drugs. Furthermore, their surfaces can be bioengineered to display various molecules, including antigenic glycans and glycoconjugate proteins (e.g., antigens and antibodies). Compared to standard delivery systems like synthetic liposomes and nanoparticles, mammalian EVs possess inherent targeting abilities. These properties enable them to cross biological barriers, such as the blood–brain barrier, making them excellent candidates for neuron-targeted drug delivery [414,415]. In addition to mammalian-derived EVs, OMVs are appealing as vaccine platforms due to their strong immunostimulatory properties. Decorating the OMV surface with antigenic glycans can help enhance their immunogenicity and overcome their naturally low immune response.

Several challenges remain in EVs research and applications. The EV isolation method currently relies on the use of ultracentrifugation or density gradient centrifugation which is cost- and time-consuming. Alternative approaches such as tangential flow filtration, size exclusion chromatography, affinity chromatography, or cyclical electrical field flow fractionation show promise in enabling efficient preparation of EVs from large volumes of culture media, and thus are paving the way for scalable production [416,417,418]. Loading yield of the designer molecules within or on the EV surface can be challenging due to low encapsulation efficiency and a lack of control over the specific molecules loaded into EVs from donor cells. The endogenous cargo of EVs typically includes a diverse array of components, such as proteins, DNA, RNA, lipids, nutrients, and metabolic waste. However, undesired cellular components cannot be excluded during the loading process. This not only lowers the loading efficacy but also poses a risk of delivering potentially harmful materials to the target [414,419]. Thus, exosome-based therapies carry potential risks, such as immune reactions, tumorigenicity, off-target effects, and gene transfer concerns. They can provoke inflammation or hypersensitivity, facilitate cancer progression, and cause unintended genetic alterations [420]. Thus, production of artificial EVs might offer a valid solution by overcoming challenges associated with endogenous EV. Bacterial EVs, particularly those derived from Gram-negative bacteria, have been shown to contain various virulence factors, including lipopolysaccharides (LPSs), virulent proteins, and toxins. These components can activate the innate immune response, potentially triggering severe inflammatory reactions that may lead to fever, septic shock, and even death. To address the issue of harmful endotoxins, mutant Gram-negative bacteria lacking LPSs, such as *E. coli* EMKV15 or ClearColi™ BL21(DE3), offer safer alternatives for drug and vaccine delivery [77,421]. EVs exhibit highly heterogeneous in terms of cargo, size, biogenesis and function mainly [422,423,424]. Improved characterization of these EVs will increase our understanding of their potential functions and applications. A multi-omic integrative approach, encompassing transcriptomics, proteomics, metabolomics, lipidomics, and glycomics is essential for dissecting extracellular vesicle (EV) content to characterize its components, understand their roles in pathogenic processes, and determine their precise composition and ratios. This comprehensive analysis is crucial for the development of artificial EVs.

It is also important to note that the clinical translation of therapeutic EVs remains challenging. First, our understanding of EVs’ biology is still limited and unwanted side effects from EVs-based therapy are possible. Second, EVs are heterogeneous in nature and production of EVs with reproducible molecular profiles is difficult. Third, challenges in large-scale EV production and cryopreservation cannot be overlooked. Fourth, as with any new drugs, current EVs particles have unclear pharmacokinetics and pharmacodynamics, which need to be investigated in detail. Finally, there is a need for an integrated system for EV preparation, purification, characterization, and quality assessment that meet the required standards for bio pharmaceuticals. In the context of regulatory guidance, both the Food and Drug Administration (FDA) and European Medicines Agency (EMA) emphasize a number of liposome characteristics that are deemed important for liposomal product performance. However, in contrast to liposome, EVs are complex vesicles derived from living cells, making it challenging to achieve the same level of specification. As a result, the community has created the Advanced Therapy Medicinal Products (ATMP) framework as a guideline for production of pharmaceutical-grade EVs. The ATMP guidelines employ a four-step risk stratification approach to identify risks, determine contributing factors, map relevant data, and assess the relationships between risks and their factors.

Looking forward, we foresee a more rapid development in the field of EV research with emphasis being placed on overcoming technical challenges in large-scale production and purification, EVs’ characterization, as well as clinical translation. Indeed, the number of preclinical and clinical studies demonstrate the potential for EV-based therapies in a number of diseases including SARS-CoV-2 pneumonia, diabetes mellitus type 1, macular holes, cerebrovascular disorders, periodontitis, neurological disease, and cancer. Finally, integrated multi-omics analysis with artificial intelligence (AI)-based data analysis and interpretation is an emerging sub-field that will further facilitate our understanding of the EVs’ biology as well as realization its biomedical applications.

## Figures and Tables

**Table 1:** Summary of EV isolation techniques.

Techniques	Description	Advantages	Disadvantages	References
Traditional techniques				
Ultracentrifuge (UC)	Separation by size and density through sequential centrifugation	Well-established protocol Easily accessible instrumentation	Cross contamination between fractions Time and energy consuming Challenging to scale up Loss of lipids and apoproteins during high-speed centrifugation	[184,185,186,187,188,189,190]
Size-exclusion chromatography (SEC)	Separation of EVs from contaminating proteins based on size differences	Efficient removal of small contaminants Structural/functional integrity of EVs are preserved	May need additional steps to remove large non-EV protein Variable efficiency, depending on sample volume/EV concentration Limited clinical applicability	[191,192,193,194,195,196,197,198]
Ultrafiltration (UF)	Size-based separation of EV using membranes with specific pore sizes or properties	Biophysical property and functionality preservation Easy to couple with other methods such as SEC (UF-SEC), which improve yield and purity	Challenging for isolation of small EVs Low purity when performed as standalone method Need for optimization of membrane pore size to reduce membrane clogging	[197,199,200,201,202]
Precipitation	Use chemicals to induce precipitation, followed by low-speed centrifugation to isolate EVs pool	High EVs yield with preserved biological activities and physical properties Particularly useful for EVs omics analysis Scalable and cost-effective for processing large sample volumes	Typically requires additional purification steps to remove precipitating agents High abundance of co-precipitated proteins which could complicate proteomics analysis	[203,204,205,206,207,208,209]
Emerging techniques				
Tangential flow filtration (TFF)	Ultrafiltration technique but with sample flows in parallel to the membrane	Minimizes membrane fouling Controllable shear force/rate Applicable for continuous operation	High operational complexity and initial capital investment Concentration polarization can occur, and this reduces filtration performance over time	[210]
Asymmetrical flow field-flow fractionation (AsFlFFF)	A size-based separation technique based on diffusion coefficients for fractionating EVs	Large analysis range Particularly useful in fractionating EV subpopulations Minimal shear forces	Potential for EVs loss due to excessive focusing time Sample-dependent performance	[183,211,212,213,214]
Charge-based techniques (exchange chromatography, electrophoresis, and dielectrophoresis)	Using surface charge on EVs for separation via electrostatic interaction or electrophoretic mobilities	Efficiently removes biomolecule contaminants from EVs due to differences in charge density	Not suitable for isolating EVs subpopulation High concentration ions used for elusion can disrupt EVs’ structures and/or functions	[214,215,216,217,218,219,220]
Affinity-based techniques	Isolation technique using specific natural or engineered ligands on EV surface	High specificity for EV subpopulation Readily adaptable for different modes of purification (batch or continuous)	Indistinguishable between proteins in solution or EV surface Specific antibodies or binders may not be available Elusion step must be optimized for each binder	[182,185,220,221,222,223,224]

**Table 2:** Summary of EVs’ characterization techniques.

Techniques	Description	Advantages	Disadvantages	References
Traditional method				
Dynamic light scattering (DLS)	Analysis of light scattering from Brownian motion to determine particle size	High accuracy for monodisperse samples ranging from 1 nm to 6 μm	Diminished accuracy when applied to polydisperse EVs populations	[236,237,238]
Transmission electron microscopy (TEM)	Visualization of EVs using electron microscope	High-resolution imaging of EVs morphology down to 1 nm Can be coupled with techniques such as cryo-TEM, immuno-gold labeling to detect EV surface proteins Adaptable for detecting EVs in physiological liquid (liquid-cell TEM)	Sample fixation requirement Standard TEM vacuum can damage specimen and introduce artifacts Necessitates sophisticated and specialist equipment	[236,239,240,241,242,243,244,245]
Nanoparticle Tracking Analysis (NTA)	Measure EVs’ size distributions and concentrations using real-time tracking of individual particles suspension (Brownian motion)	Applicable for the following particles as small as 30 nm (small EVs) Can be coupled with fluorescence-based phenotyping of EVs	Limited accuracy for very small EVs due to low light scattering or very large EVs due to their very slow movement Does not provide information about molecular composition	[235,246,247,248,249]
Flow cytometry	Microfluidic-based detection of EVs morphology and/or fluorescent signals	Rapid profiling and sorting of EV subpopulations Allow determination of EVs’ origin through surface marker labeling Can be coupled with image analysis	Detecting small EVs, such as exosome is challenging Shear force from microfluidic chambers can compromise EV integrity	[235,241,250,251,252,253]
Omics approach				
Proteomics	Comprehensive mass spectrometry-based profiling of total proteome in particular EVs	In-depth analysis of EVs’ proteomic components Can be used to determine EVs’ origin, surface markers Allow for use of EVs in disease diagnostic	Challenging to obtain high-quality data Complicated sample processing and data analysis Mass spectrometry analysis is not yet fully optimized for all PTMs	[254,255,256,257,258,259,260,261,262,263,264,265,266,267,268,269,270,271,272,273,274]
Transcriptomics	Profiling total transcripts (mainly mRNA) using RT-PCR and NGS approach Providing insights into cellular state and serving as potential biomarkers during pathogenesis	Rather matured discipline with several robust protocols and commercial products available Provide insight into cell state through differential expression profile Possible to profile transcript from single EVs	Transcriptomics and proteomics profile does not always correlate well Missing influence of PTMs on proteins, e.g., transcriptomics cannot predict glycosylation outcomes Challenging to validate non-coding RNAs	[275,276,277,278,279,280,281,282]
Genomics	Sequencing of DNA within EVs	Rather matured discipline Genetic materials in EVs can be used as parental cell surrogates during disease progression	Challenge in data analysis and interpretations Exome variants can be misinterpreted	[283,284,285,286,287]
Lipidomics	Mass spectrometry-based approach to profile total lipid compositions and abundance within EVs	Reveal roles of lipids in various pathogenicity including diabetes and inflammation	Sample complexity with diverse structures in a small mass range (3–900 Da)	[288,289,290,291,292,293]
Metabolomics	Mass spectrometry-based or nuclear magnetic resonance (NMR)-based analysis to profile total small molecule metabolites within EVs	Promising approach to identify biomarkers for monitoring disease, aging, or drug developments	A need to purify or fractionate sample prior MS analysis to reduce sample complexity Large amount of data and challenges in data analysis	[294,295,296,297,298]
Glycomics	Mass spectrometry- and/or carbohydrate binding molecules-based profiling of glycans and glycoconjugates on the surface and within EVs	Accurately identify glycans and glycoform-specific conjugates using diagnostic ions Reveal roles of glycans during homeostasis, development, and disease progression	Glycans are extremely heterogeneous and typically are present at minute quantities Tedious and time-consuming sample preparation Lack of automated data analysis tools	[299,300,301,302,303,304,305,306,307]

**Table 3:** Licensed and clinically developed vaccines based on outer membrane vesicles adapted from Micoli et al. [413].

Vaccine	Pathogen	Company
Licensed vaccine		
Bexsero (4CMenB)	*Neisseria meningitidis* serogroup B	GSK (Siena, Italy)
MenZB (NZ dOMV)	*Neisseria meningitidis* serogroup B	Novartis Vaccine and Diagnostics (Siena, Italy) ^b^ and National Institute of Public Health (Oslo, Norway)
VA-MENGO-BC	*Neisseria meningitidis* serogroup B	Finlay Institute (Havana, Cuba)
Norway MenBVAC	*Neisseria meningitidis* serogroup B	Norwegian Institute of Public Health (Oslo, Norway) ^a^
PedvaxHib (PRP-OMPC)	*Haemophilus influenzae* type b	Merck Co. (Rahway, NJ, USA)
Procomvax/Comvax (PRP-OMPC and hepatitis B)	*Haemophilus influenzae* type b and hepatitis B	Merck Co. (Rahway, NJ, USA) ^a^
Vaxelis (diphtheria and tetanus toxoids, acellular pertussis, inactivated poliovirus, PRP-OMPC and hepatitis B)	Diphtheria, tetanus, pertussis, poliomyelitis, *H. influenzae* type b and hepatitis B	Merck Co. (Rahway, NJ, USA) and Sanofi Pasteur (Lyon, France)
Phase I/II clinical trial		
altSonflex1-2-3	*Shigella*	GSK (Siena, Italy)
Avacc	COVID-19	Intravacc (Bilthoven, Netherlands)
iNTS-GMMA	Invasive non-typhoidal *Salmonella*	GSK (Siena, Italy)
N/A	*Neisseria gonorrhoea*	GSK (Siena, Italy)

^a^ Marketing authorization was not renewed by the market authorization holder (Procomvax). ^b^ No longer licensed, NVD was taken over by GSK.

## Abbreviations

AD	Alzheimer’s disease
ApoEVs	apoptotic bodies from programmed cell death or apoptosis
BBB	blood–brain barrier
BEVs	bacterial extracellular vesicles
CAR	chimeric antigen receptor
CDC	cardiac-derived cells
CHO	Chinese hamster ovary
CMVs	cytoplasmic membrane vesicles
CPCs	cardiac progenitor cells
CVD	cardiovascular disease
DCs	Dendritic cells
DC-SIGN	dendritic cell-specific intercellular adhesion molecule-3-grabbing non-integrin
DLS	dynamic light scattering
DOX	doxorubicin
ELVs	exosome-like vesicles
EOMVs	explosive outer-membrane vesicles
ESCRT	endosomal sorting complex required for transport
EVs	extracellular vesicles
GMMAs	Generalized Modules for Membrane Antigens
glycOMVs	glycoengineered OMVs
GTs	glycosyltransferases
ILVs	intraluminal vesicles
iPSC-EVs	induced pluripotent stem cells
LPSs	lipopolysaccharides
MSC	mesenchymal stem cell
NSCLC	non-small-cell lung cancer
NSC-exos	neuronal stem cell-derived exosomes
OIMVs	outer–inner membrane vesicles
OMVs	outer membrane vesicles
PAMPs	pathogen-associated molecular patterns
PEG	polyethylene glycol
SEC	size-exclusion chromatography
SPT	single-particle tracking analysis
TEM	transmission electron microscopy
